# Research on liver cancer segmentation method based on PCNN image processing and SE-ResUnet

**DOI:** 10.1038/s41598-023-39240-0

**Published:** 2023-08-07

**Authors:** Lan Zang, Wei Liang, Hanchu Ke, Feng Chen, Chong Shen

**Affiliations:** 1 State Key Laboratory of Marine Resource Utilization in South China Sea, Hainan University, Haikou, 570228 China; 2 School of Information and Communication Engineering, Hainan University, Haikou, 570228 China; 3 School of Electronic Science and Technology, Hainan University, Haikou, 570288 China; 4 Department of Radiology, Hainan General Hospital (Hainan Affiliated Hospital of Hainan Medical University), Haikou, 570216 China

**Keywords:** Cancer, Computational biology and bioinformatics, Diseases, Oncology

## Abstract

As one of the malignant tumors with high mortality, the initial symptoms of liver cancer are not obvious. In addition, the liver is the largest internal organ of the human body, and its structure and distribution are relatively complex. Therefore, in order to help doctors judge liver cancer more accurately, this paper proposes a variant model based on Unet network. Before segmentation, the image is preprocessed, and Pulse Coupled Neural Network (PCNN) algorithm is used to filter the image adaptively to make the image clearer. For the segmentation model, the SE module is used as the input of the residual network, and then its output is connected to the Unet model through bilinear interpolation to perform the down-sampling and up-sampling operations. The dataset is a combination of Hainan Provincial People's Hospital and some public datasets Lits. The results show that this method has better segmentation performance and accuracy than the original Unet method, and the dice coefficient, mIou and other evaluation indicators have increased by at least 2.1%, which is a method that can be applied to cancer segmentation.

## Introduction

Liver cancer is the second leading cause of cancer death in the world, and hepatocellular carcinoma (HCC) accounts for about 90% of all primary liver cancers^1^. Metastatic hepatic carcinoma is a primary malignancy that metastasizes to the liver from other parts of the body and forms single or multiple cancer foci in the liver, requiring treatment of both the primary lesion and the metastatic foci. Therefore, the main thing we studied is the characteristics of primary liver cancer.

The onset of primary liver cancer is closely related to the following factors: (a) Viral hepatitis, the most common being the hepatitis B virus. Statistical results show that people who have suffered from hepatitis B have a 10 times higher rate of liver cancer than people who have not had hepatitis B, and hepatitis, cirrhosis, and liver cancer are regarded as an evolving trilogy; (b) Alcohol, one of the characteristics of liver cancer is slow metabolism, and drinking alcohol is easy to cause liver cells to decompose and metabolize fatty acids, which in turn causes liver fibrosis, cirrhosis, and liver cancer; (c) Eating habits are the main cause of the frequent occurrence of liver cancer in tropical regions like Hainan^2,3^. The abundant precipitation and high temperature weather in the tropics have created extremely favorable conditions for the pollution of aflatoxin. Aflatoxin has a significant positive correlation with the incidence of liver cancer. HCC evolves gradually on the basis of chronic liver disease or liver cirrhosis, and it is a multi-step cancer. Early HCC is the initial stage of HCC formation, mostly 1.0–1.5 cm in diameter, indistinct nodules. Among them, dysplastic nodule (DN) is a precancerous lesion and an important basis for early diagnosis and identification.

When processing cancer images, imaging techniques such as computed tomography (CT), magnetic resonance imaging (MRI) and ultrasound (US) are usually used. Among them, CT and MRI are more sensitive in detecting early liver cancer ^4–6^. Medical images provide doctors with rich auxiliary information to help them make judgments. As an important part of medical image processing, image segmentation is of great significance for accurate identification, detailed analysis, reasonable diagnosis, prediction and prevention of cancer^7–9^. Therefore, how to complete image segmentation is the key point we need to pay attention to.

Due to the influence of external machines on the shape, size, and position presented by medical images, there are significant differences in grayscale, unclear boundaries, and more importantly, complex texture changes, making it difficult to control the accuracy of segmentation. Traditional segmentation techniques such as threshold method, region growth method, edge detection method, etc. may have poor accuracy, efficiency, and robustness. However, segmentation methods based on convolutional neural networks and deep learning development can utilize high-dimensional features without manual intervention, but may overlook issues such as image pixels, noise, and segmentation results deviation caused by fuzzy tumor boundaries. In order to solve the above problems, based on the Unet model, this paper takes SE module as the input of the residual network, connects its output to the Unet model through bilinear interpolation, and conducts down sampling and up sampling operations respectively. Before segmentation, in order to avoid the impact of noise and unclear boundaries in the image itself, the image is preprocessed using the PCNN algorithm for adaptive filtering, making the image ^12–14^ clearer and achieving better results in subsequent segmentation.

Our research contributions are as follows:

1. An image preprocessing module was proposed, which utilizes PCNN to adaptively filter the image and obtain the normalized image;
2. The SE Resnet module was proposed, with the SE module as the input to the Resnet module, weighting the change information and calculating the information that can recognize image features;
3. In order to solve the problems of poor segmentation and unclear sample recognition, a segmentation method based on PCNN processing and SE ResUnet was designed, which effectively solves the problems of unclear and incomplete images.

The rest of this paper is organized: the literature review in the second part, the third part describes the algorithm flow and relevant models of this paper, the fourth part is the results and discussion, and the fifth part is the conclusion.

## Related works

The early methods of medical image segmentation are mainly completed by using edge detection, shape model, active contour and other methods, so the traditional segmentation methods include threshold method, region growth method, edge detection method, clustering method and so on. Some researchers will first preprocess the image to lay a better foundation for subsequent segmentation processing^10,11^. Liu et al. briefly described the segmentation technology of medical images, and also summarized the traditional methods based on boundary, threshold, region growth, statistics, graph theory, information theory and so on in detail^15^. In addition, Gao et al. proposed that the image histogram can be converted into a wavelet system using the principle of wavelet transform, and then the threshold value can be determined by combining the segmentation conditions to divide the tumor region^16^. Ren et al. systematically reviewed the current situation of medical segmentation, introduced four traditional segmentation methods such as threshold method and region method, and proposed an automatic segmentation method based on deep learning, which plays an important role in the medical field^17^.

As a prominent representative of deep learning technology, convolutional neural network has gradually emerged as a series of algorithms based on CNN in the field of medical image segmentation with its rapid development in various fields, and the advantages of deep learning methods have also helped doctors more easily distinguish different organs or lesions and give the most accurate treatment plan. Wang et al. elaborated the definition of graph in graph depth learning algorithm and the basic structure and working principle of GCN, and summarized the progress and future challenges of graph depth learning algorithm in medical image segmentation^18^. Based on machine learning and deep learning technology, Painuli summarized breast cancer, lung cancer, liver cancer, skin cancer, brain cancer, pancreatic cancer, and compared various advanced technologies to test the key performance indicators such as area under the precision curve and sensitivity^19^. Peng et al. classified and summarized three methods based on full convolution neural network, Unet network and its variants, as well as specific design ideas, and proposed relevant evaluation indicators and data sets for research in the field of medical image segmentation, and pointed out the problems that need to be studied in the future^20^. Gul et al. summarized the existing deep learning methods for liver segmentation and detection, compared with the performance indicators of early segmentation methods, and covered the improvement of different deep learning architectures for CT and MRI segmentation tasks^21^. Ghaznavi proposed a method of combining residual attention with Unet, the live cells of HeLa line are segmented under the light transmission microscope, and the watershed segmentation method is used to separate the cells to avoid over-segmentation and extract the important information of cells^22^. Wang et al. added ResNet's direct mapping structure to the original path of Unet, and achieved good segmentation performance on the placenta dataset, which can achieve more accurate segmentation^23^. Xie et al. proposed dynamic adaptive residual network (DAR-Net) to remove irrelevant pixels and optimize the liver boundary and texture using conditional random fields to improve the segmentation accuracy^24^. Jiang et al. proposed the Unet++ model based on Attention Hybrid Connection Network (AHCNet) to achieve faster network convergence and accurate semantic segmentation. Pankaj proposed the Unet model based on attention channel, and used cross validation K5 to help segment bright and blurred images^25^. Because the continuous pooling and downsampling operations of Unet will lead to the loss of some spatial information, Sun proposes a U-type context residual network (UCR-Net), which can capture more context and efficient information and recover more advanced semantic features^26^. Liu et al. proposes a deep neural network with Res-Net structure, which combines the residual network (ResNet) and Unet to build a code-decode network, deepen the layers in the network, and save more details in low-quality images^27^. Beeche et al. integrate a dynamic acceptance domain module and a fusion upsampling module into the Unet architecture to form a super Unet, which can be applied to image segmentation of blood vessels, gastrointestinal tract, skin diseases, etc.^28^. Lv proposes an improved liver CT segmentation method based on iRes-Unet. In view of the shortcomings of Unet in performance, BN layer is introduced to eliminate the covariate shift within the network, improve the generalization and convergence speed, and the Residual-module is introduced to enhance the edge thinning^29^. Li first uses Gaussian filter to de-noise the image, then processes the CT image through ecological operation, and finally uses Res Unet model to improve the segmentation loss and improve the segmentation accuracy and speed^30^. Lin Yuchun et al. studied the performance of U-Net in fully automatic positioning and segmentation in magnetic resonance (MR) images, and proved that Unet network can perform more accurate segmentation in diffusion-weighted MR images^31^. Zhang et al. designed an embedding mechanism based on modal sensing features to complete the segmentation task by inferring the important weight of modal data in the network learning process^32^. Zhang et al. integrated the Inception-Res module and the densely connected convolution module into the U-net architecture, which increased the width and depth of the network without additional parameter assistance^33^. Ange et al. replaced the skip connection with the residual module, and designed a new architecture—DC-UNet. At the same time, the valley similarity was used to replace the Jaccard similarity for gray image comparison^34^. Zheng et al. added a pure converter to the FCN and added receptive fields by expanding/empty convolution or inserting attention modules. Each layer models the global context, providing a powerful segmentation model^35^. Feng et al. proposed a context pyramid fusion network (CPFNet) to dynamically fuse multi-scale context information in advanced features, which has strong competitiveness in different tasks^36^. Li et al. proposed a new end-to-end hybrid dense connection UNet (H-DenseUNet), which can be jointly optimized by using the hybrid feature fusion layer. It is composed of 2-D DenseUNet for effectively extracting features in slices and 3-D corresponding for hierarchical aggregation volume context, with good segmentation effect^37^. Huang et al. established a new rule combination strategy by using diagnostic rules to construct the component classifier of Adaboost algorithm, which can solve the classification problem of different feature spaces (PC-DFS) and has good prediction performance^38^. Literature ^39^ summarized more than 90 related papers, and compared the evaluation indicators, showed that the hybrid model performs better in liver disease and lesion segmentation tasks, and reviewed the existing algorithms and their performance in various image analysis tasks. Therefore, we have summarized and compared some of the algorithms mentioned in the literature, as shown in Table 1.

**Table 1:** Comparison of the performance of various algorithms mentioned in the literature.

Method	Datasets	IoU	Dice	Acc	RVD	PPV
Residual attention U-Net^22^	HeLa cells	0.9530	0.9758	0.9600	–	–
RU-Net^23^	Placenta	–	0.9547	–	1.32%	–
UCR-Net^26^	Femoropopliteal artery stent	0.9481	0.9732	0.9992	–	–
improved U-net^27^	Nailfold capillary	–	0.9766	0.9172	–	–
Super U-Net^28^	ISIC dataset	–	0.877	0.956	–	0.963
DENSE-INception U-net^33^	Retinal vessels	–	0.9582	0.9657	–	–

## Method

### Experimental design

**Figure 1:**
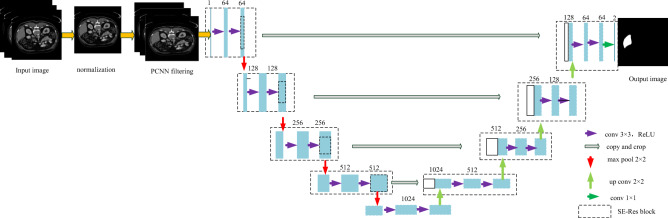
Overall process framework.

Both MRI and CT images inevitably experience degradation in image clarity and noise during production, transmission, and storage. Therefore, this article first preprocesses all images in the dataset. First, scale the image scaling to 256 × 256 pixels, and normalize the image using histogram based intensity, while unifying the data size to 256 × 256 × 16. Then, the image is input into the PCNN model for adaptive filtering, and the filtered image is output to obtain a clear image. The filtered image is input into the improved U-shaped network structure, which mainly consists of three parts: encoder, decoder, and skip connection structure. It is a typical fully convolutional network with encoding and decoding structure. This article is based on a typical U-shaped network structure and introduces the SE-Resnet network module to segment the filtered image. In the SE-Resnet module, the SE module is used as the input of the residual block to weight the change information and calculate the information that can recognize image features. The residual block can increase the number of network layers and better integrate the semantic information of the image. The SE-Resnet module is inserted into the Unet model in the form of bilinear interpolation, which can not only use the number of channels in the residual module to reduce the training parameters and computation, but also integrate the SE attention mechanism to further improve the performance of feature details, and play a certain role in improving the recognition rate and accuracy in the segmentation process. The overall flowchart is shown in Fig. 1. In this design, the image segmentation process adopts a Unet network as the basic network structure, replacing the sub modules in the network with SE-Resnet modules to enhance the correlation of feature information. The feature information processed by the SE-Resnet module in the encoding network can obtain the weight information of different pixels in the feature map, and train and calculate the network based on the weight information to reduce the interference of redundant information. This model can appropriately fit the complex correlations between channels, reducing the number of parameters and computational burden caused by the increase in network depth.

The advantage of this is that the SE module, as the input of the residual network, can capture more feature information in the Unet model, help to accurately detect and segment the image data, at the same time, it can effectively reduce the complexity and memory occupation of the model, inhibit the unimportant features in the Feature Map, and make the model more generalized.

### Image preprocessing

**Figure 2:**
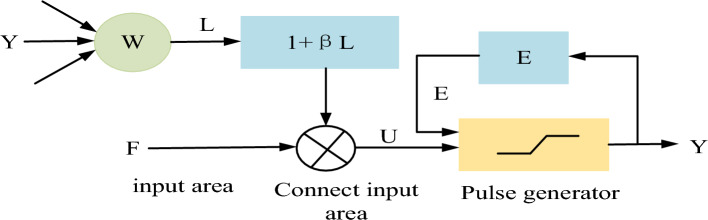
PCNN model.

Due to the impact of machine operation on images, patients may experience unconscious body shaking, resulting in blurred images. In order to achieve better segmentation results, we use Pulse Coupled Neural Network (PCNN) to denoise the image before segmentation, in order to obtain a clearer image. PCNN is proposed based on the phenomenon of synchronous pulse emission from animal cerebral cortex ^12–14^. It is a dynamic nonlinear neural network composed of several interconnected neurons, including receiving input domain, nonlinear connection modulation domain, and pulse generation domain, as shown in Fig. 2. The receiving domain receives stimulus inputs from adjacent neurons and the outside world, consisting of a linearly connected input channel $L$ and a feedback input channel $F$. The output of each neuron has only two states, ignition or non ignition.

When PCNN is used in image processing applications, it is a single-layer two-dimensional locally connected network, where neurons correspond to image pixels one by one and are also interconnected with adjacent neurons. PCNN does not require parameter training. Therefore, for the input image, uniformly set the parameters:$\beta = 0.5$, $V_{E} = 2.0$, $\alpha_{E} = 0.3$, and the grayscale value of the pixel points in the image is 0–255. The initial threshold is set to the maximum grayscale value in the image. The threshold of each neuron in the first iteration is the maximum value. As the iteration progresses, the threshold gradually decreases. The points with higher brightness in the image correspond to neurons firing and outputting 1, while other neurons outputting 0. Then perform 3 on pixels with PCNN output of 1× By using local morphological filtering, these bright noise points can be removed. For darker noise points in the image, the image after removing the brighter noise points can be subjected to inverse processing, so that the darker points can obtain the denoised image. Among them, the use of morphological opening and closing operations to remove smaller highlights is defined by the cascade of grayscale expansion and corrosion. The open operation can be obtained by calculating the union solution of the translations of all structural elements that can be filled in the image, that is, marking the position of each filling structural element, and then calculating the union when the far point of the structural element is translated to each marked position, to obtain the open operation structure. Due to the duality of expansion and corrosion, open and closed operations can also obtain corresponding duality.

1 $$F_{{{\text{ij}}}} \left[ n \right] = \exp \left( { - \alpha {}_{F}} \right)F_{{{\text{ij}}}} \left[ {n - 1} \right] + V_{F} \sum {m_{ijkl} } Y_{kl} \left[ {n - 1} \right] + S_{ij}$$

2 $$L_{{{\text{ij}}}} \left[ n \right] = \exp \left( { - \alpha {}_{L}} \right)L_{{{\text{ij}}}} \left[ {n - 1} \right] + V_{L} \sum {m_{ijkl} } Y_{kl} \left[ {n - 1} \right]$$

3 $$U_{{{\text{ij}}}} \left[ n \right] = F_{{{\text{ij}}}} \left[ n \right](1 + \beta L_{{{\text{ij}}}} \left[ n \right])$$

4 $$Y_{ij} [n] = 1\,if\,U_{ij} [n] > \theta \,or\,0$$

5 $$E_{ij} \left[ n \right] = {\text{exp}}\left( { - \alpha_{E} } \right)E_{ij} \left[ {n - 1} \right] + V_{E} Y_{ij} \left[ {n - 1} \right]$$

where *S* is the input excitation, representing the pixel gray value of (*i*, *j*). *F* is the input of neurons, *L* is the connection input, *U* is the internal activity item of neurons, *m, W* is the internal connection matrix, *Y* and *0* correspond to the output and dynamic threshold of neurons respectively. PCNN is used for image denoising and enhancement. Using PSNR (Peak Signal to Noise Ratio) as the comparison standard for experimental results.

6 $$PSNR = {1}01\log_{10} \frac{{255^{2} }}{{\frac{1}{MN}\sum\limits_{i = 1}^{M} {\sum\limits_{j = 1}^{N} {\left( {f(i,j) - f^{\prime}(i,j)} \right)^{2} } } }}$$

where $f(i,j)$ is M×N original image pixel grayscale value, $f^{\prime}(i,j)$ is A is the grayscale value of the M×N image after filtering the noisy image, M and N represent the number of rows and columns of the image, respectively.

**Figure 3:**
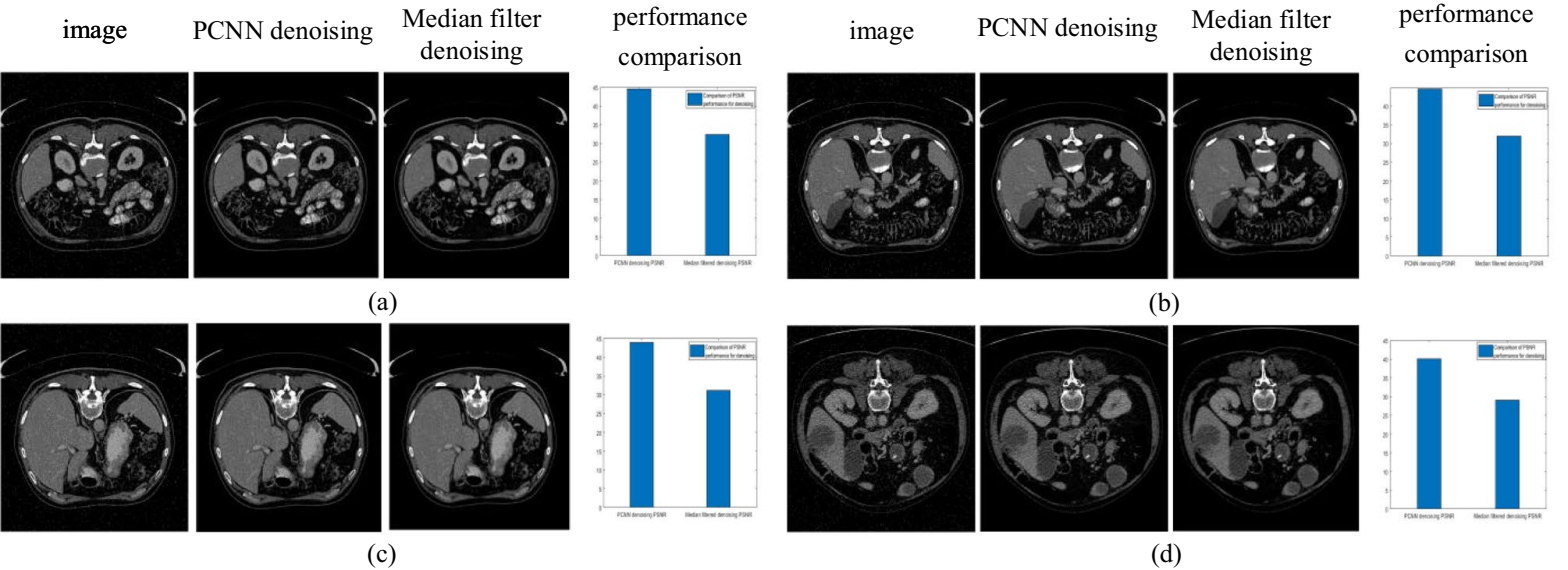
Comparison of filtering results.

Comparing the PCNN algorithm with the most commonly used median filtering algorithm, it was found that the image processed by PCNN is clearer than the original image and better reflects detailed features and image information than the median filtered image. And comparing the PSNR performance of the two algorithms, it was found that the PCNN algorithm has a larger PSNR and the filtered results are closer to the original image. The results are shown in Fig. 3.

### Residual network

**Figure 4:**
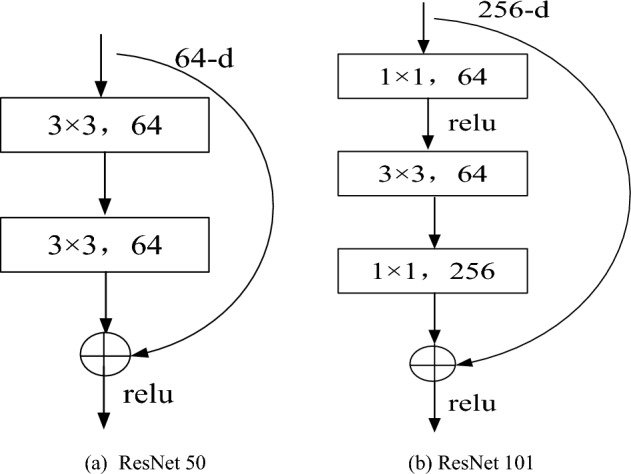
Two structures of residual block.

Residual Network is composed of identity mapping and residual part. Its architecture is derived from residual unit, so each layer contains two branches. One is to take the output of the upper layer as the input of this layer, and the other is to take the output of the upper layer as the input of this layer after some transformation^23,24^. The inputs of the two branches will be added and summed to form the residual learning unit. There are two kinds of residual block structures. One is designed with the full 3 × 3 convolution layer in the VGG structure, and the output channels of these two convolution layers are the same, as shown in Fig. 4a. The other structure is successively composed of three convolution layers (1 × 1, 3 × 3, 1 × 1) in series, as shown in Fig. 4b. The residual network adds the output and input of multiple convolutional cascades to extract the features of the image^26^. It innovatively adopts the "shortcut connection", which can skip one or more layers and make the input results directly transmitted to the bottom layer. The calculation formula is as follows:

7 $$y = f\left( x \right) + x$$

where *x* is the input result, *y* is the final output result, and *f* (*x*) is the output result of the hidden layer in the network.

Because the number of layers of the residual network is different, the structure application is different. Resnet50 is selected in this paper, so the residual structure needs to be shown in Fig. 4b. It adds a 1 × 1 convolution layer dimension reduction before calculation, and carries out a 1 × 1 convolution recovery after 3 × 3 convolution operation, so as to reduce the amount of calculation. The innovation of residual network is that it uses jump connection to connect output data back to input data, thus effectively eliminating signal loss^28^. In addition, it also uses the superimposed network layer, which makes the deep neural network more robust and can effectively calculate the back-propagation gradient.

### SE attention

**Figure 5:**
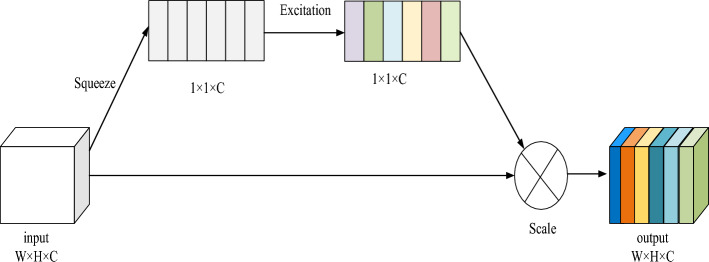
SE module.

The core idea of SE (squeeze and excitation) attention mechanism is to ignore irrelevant information and only extract regions of interest, which includes three operations: sequence, exclusion, and scale. Sequence uses a global average pooling to solve the mean value of all elements on each channel, that is, map the input feature map to the same low-dimensional space as the number of feature channels^30^. Excitation is the core operation of SE. It uses a full connection layer to map the features of low-dimensional space to high-dimensional space, and readjust the weight of each feature map. Finally, all weights are combined to form the distribution of feature importance. SE module plug and play means that it can strengthen the channel characteristics of the input characteristic map, and the final SE module output does not change the size of the input characteristic map. The structure of SE module is shown in Fig. 5.

SE attention model is a very popular model in deep learning. It can provide individual weights for each layer of deep network, so as to realize the distribution of feature importance. SE attention model can also improve feature selection, balance intra-class and inter-class proportions, and adjust the importance of features, all of which help to improve the performance of the model^27^. The main process includes:

- *Vectorization*: Convert each word in a sentence into a numerical vector to better express its meaning.
- *Matrix multiplication*: calculate the vector of each word in the sentence by matrix multiplication to get a self-attention "head", which is used to express the relationship between different words.
- *Calculate attention*: calculate the attention between each word and other words by calculating the similarity between the vector of each word and the vector of other words.
- *Connection result*: connect all the self-attention heads together to form the final sentence vector to express the meaning of the sentence.

### SE-Resnet network

**Figure 6:**
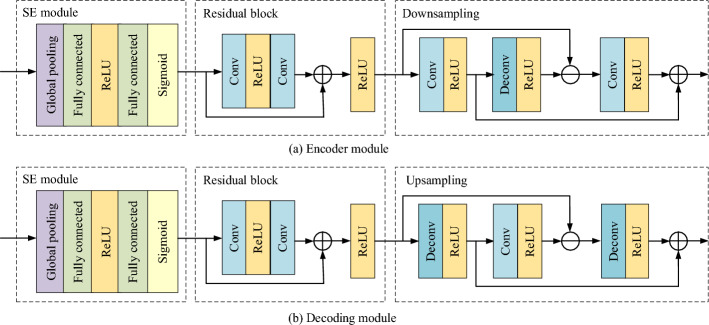
Encoding and decoding of segmentation algorithms.

In order to avoid the gradient disappearance and over-fitting phenomenon of U-Net network with the deepening of network structure, the U-Net network model is improved. In this paper, the residual network module and attention mechanism are introduced into the model. The two most important parts in the SE module are the fully connected layer and feature multiplication fusion. Assuming input image H × W × C is stretched to the dimension of 1 × 1 × C through global pooling and fully connected layers, then multiply by the original image and assign corresponding weights to each channel to achieve feature fusion. The residual structure better preserves the spatial information of features, overlays deep features with low-level features to obtain enhanced feature information, and bypasses unnecessary multi-layer networks to directly input results, effectively improving the segmentation ability of the model. The structural connection of the SE-Resnet network is that the SE attention module is first connected to the input end of the residual block, that is, the SE attention module is used as the input module of the residual block, and the SE attention adopts the weighted attention mechanism, which can weight the change information from the image and calculate the information that can recognize the image characteristics. Then, the output of the residual block is connected to the Unet model by bilinear interpolation for down-sampling and up-sampling operations, as shown in Fig. 6.

In order to reduce the training amount of parameters, SE mechanism is introduced on the basis of Resnet. The core module of SE ResNet is the combination of squeezing and excitation blocks (SE blocks) and Resnet's residual blocks to adaptively recalibrate the residual feature response at the channel level. The encoding network and decoding network each contain four layer network structures. By using a feature recalibration strategy, the network can obtain the importance of each residual feature channel, thereby enhancing useful channel features based on the importance and suppressing less useful channel features. The encoding network continuously expands its field of view through convolutional and downsampling operations, including two 3 × 3 convolutional modules and a well-defined input feature map. The decoding network restores the encoded compressed image through deconvolution and upsampling operations to form an image of the same size scale as the input, and performs pixel by pixel prediction and segmentation. In the SE Resnet module, the two 3 × 3 convolutional layers in each residual block are replaced with two 1s × 1 attention layer and add a residual connection to ensure the depth of the network. At the same time, inserting a residual structure between the input layer and the output layer can effectively avoid the Vanishing gradient problem with the increase of network depth. At the same time, the SE module introduces a global pooling layer, which can effectively capture and utilize feature information and improve model performance. The residual structure better preserves the spatial information of features, overlays deep features with low-level features to obtain enhanced feature information, and bypasses unnecessary multi-layer networks to directly input results, effectively improving the segmentation ability of the model.

First of all, set *X* as input data, *Y* as output data, *H*_*1*_ as one layer of residual block, *H* as two layers of residual block, *F* and *F*2 as multi-head attention mechanism, and *K* and *D* as the number and depth of multi-head attention mechanism.

Then, the structure of a layer of residual blocks and SE attention modules can be expressed as:

8 $$Y = H_{1} \left( X \right) + F_{1} \left( {X,k,d} \right)$$

The structure of multi-layer residual block and SE attention module can be expressed as follows:

9 $$Y = H_{1} \left( {H_{2} \left( X \right)} \right) + F_{1} \left( {H_{2} \left( X \right),k,d} \right) + F_{2} \left( {X,k,d} \right)$$

In the improved coding submodule, the output results of each layer are normalized first, and then activated by activation function. It is equivalent to that each subsampling operation contains two 3 × 3 convolution layers, one 1 × 1 shortcut and one 2 × 2 pooling layer. In the decoding submodule, each upsampling operation also includes two 3 × 3 convolution layers and one 1 × 1 shortcut. However, in the up-sampling operation, it needs to be fused with the structure of the corresponding coding part first, then processed and activated the output results, and finally added into the 1 × 1 convolutional network to output the final results.

## Results and discussion

### Datasets

**Figure 7:**
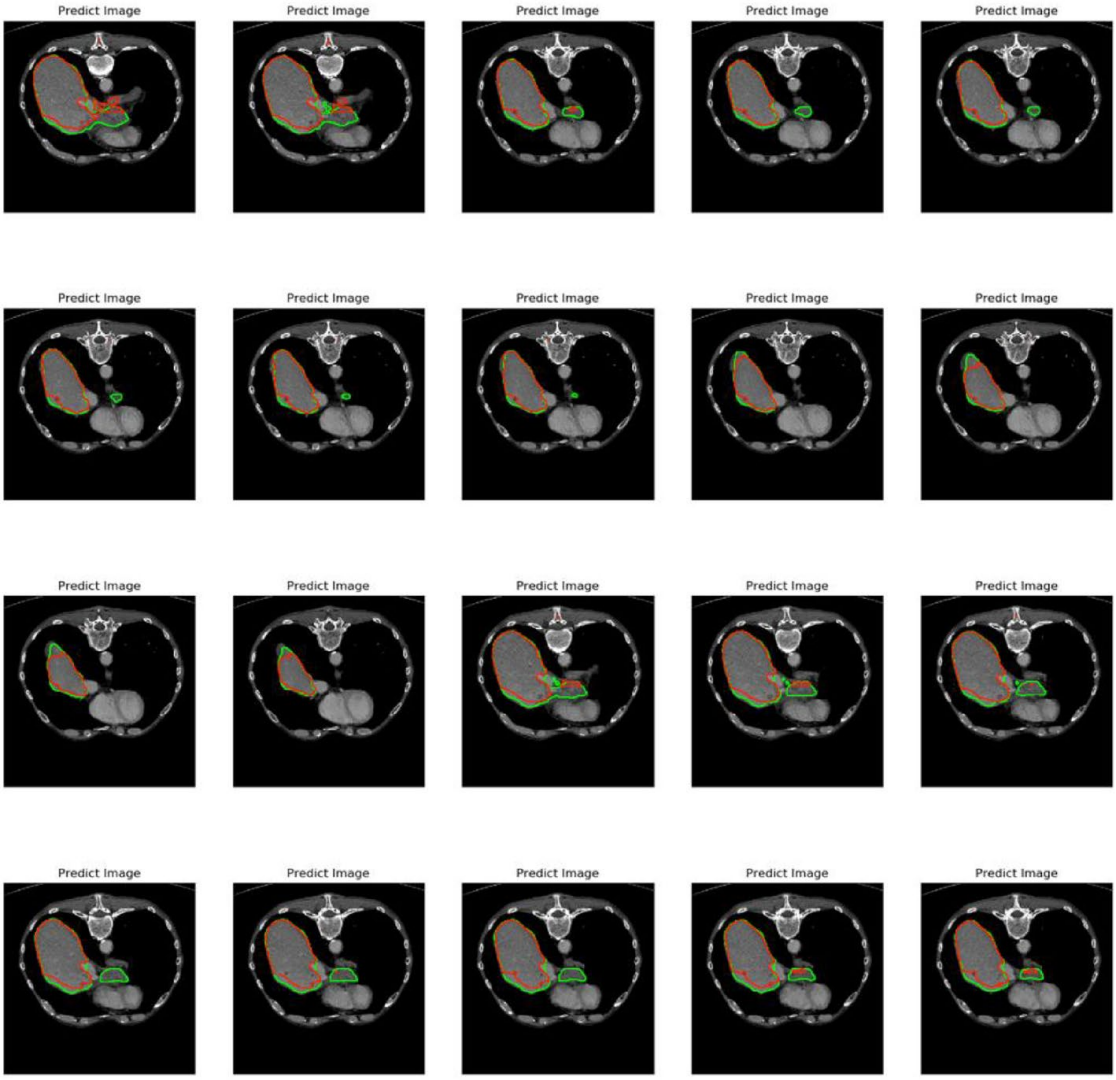
Predicted marker image.

**Figure 8:**
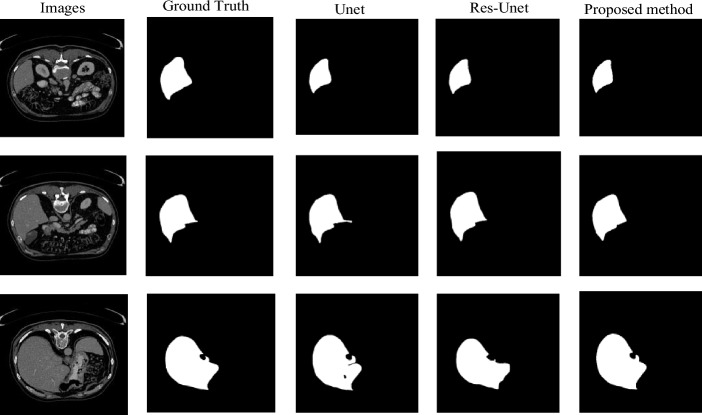
Segmentation results of three different methods.

The dataset used in this article includes desensitized data from Hainan Provincial People's Hospital and partially public dataset Lits. After processing and combining, a new dataset was collected from liver tumor patients who have been examined and treated at Hainan Provincial People's Hospital in recent years. It includes whole tumor regions, tumor core regions, and T1 and T2 weighted imaging data. Due to the fact that the collected MRI images are plain scan, there may be hundreds or thousands of corresponding MRI images for each patient. Therefore, it is necessary to find images containing liver tumor areas. We extracted 405 datasets for our study, with slice thicknesses ranging from 0.5 to 6 mm and inter plane resolution ranging from 0.58 × 0.58  to  0.95 × 0.95 mm^2^, image size 512 × 512. Before segmentation, we have already performed preprocessing such as denoising and enhancement on the image, which has been scanned earlier. Therefore, the processed image is directly input into the model for segmentation. In order to better experiment, set the parameters uniformly. The total epoch of training is 100. Gradient descent is performed every five training steps. The weight value is saved every five epochs. Channels is 3, and the Learning rate is set to 1e-4, the attenuation rate is 0.0001. Where, Fig. 7 shows the predicted labeling results under different states and angles, while Fig. 8 shows the segmentation results of several different methods. Research has found that our model's segmentation performance is closer to reality.

### Evaluating indicator

The loss function is used to qualitatively evaluate the degree of inconsistency between the predicted value and the reference value. Dice coefficient (Dice) is selected for this study, and the calculation formula is as follows:

10 $$DiceLoss = 1 - \frac{{2\left| {X \cap Y} \right|}}{\left| X \right| + \left| Y \right|}$$

where *X* is the predicted segmentation result and *Y* is the reference value.In order to better evaluate the performance of the model, three evaluation indexes, mIoU, accuracy and recall, are adopted at the same time, as shown below:

11 $$mIoU = \frac{{\left| {X \cap Y} \right|}}{{\left| {X \cup Y} \right|}} = \frac{TP}{{TP + FP + FN}}$$

12 $$Precision = \frac{TP}{{TP + FP}}$$

13 $$Recall = \frac{TP}{{TP + FN}}$$

where *TP* is the positive samples predicted by the model as positive classes, *FP* is the negative sample predicted as positive by the model, *FN* is the positive sample predicted by the model as a negative class.

**Figure 9:**
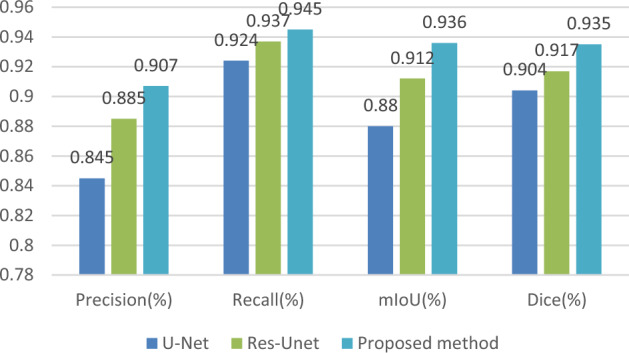
Performance comparison of evaluation indicators.

The above table shows the four evaluation indicators used to evaluate and analyze the segmentation performance of several methods. It can be seen from Table 2 that the IoU, dice and precision of the proposed method are at least 2.1% higher than other algorithms, and this method shows a good segmentation effect. Figure 9 is a comparison chart converted from the data in Table 2, which can be more intuitively displayed. In terms of performance comparison of evaluation indicators, the method proposed in this article has better results.

**Table 2:** Segmentation results of different segmentation algorithms.

	Precision (%)	Recall (%)	mIoU (%)	Dice (%)
U-Net	0.845	0.924	0.88	0.904
Res-Unet	0.885	0.937	0.912	0.917
Proposed method	0.907	0.945	0.936	0.935

**Figure 10:**
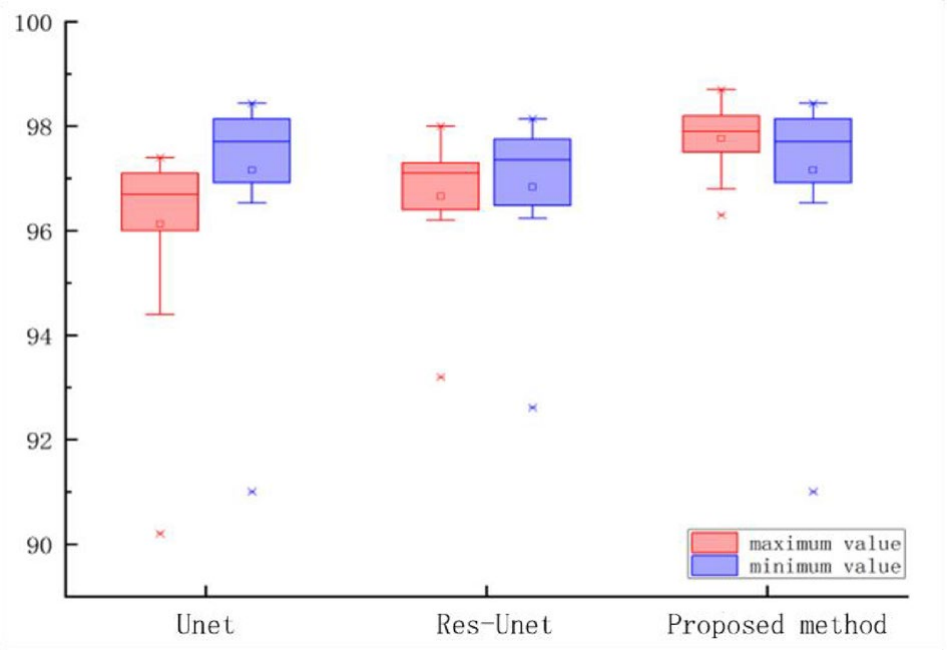
Box diagram of IoU.

Figure 10 shows the box diagram of IoU. From the figure, it can be seen that the red box line represents the maximum group value for each model, while the blue box line represents the minimum group value. Among them, the middle line of the box is the median of the data, representing the average level of overall data training. Comparing the three models, it was found that the My Net model has a relatively stable effect in terms of data distribution, overall data variance, and standard deviation.

**Figure 11:**
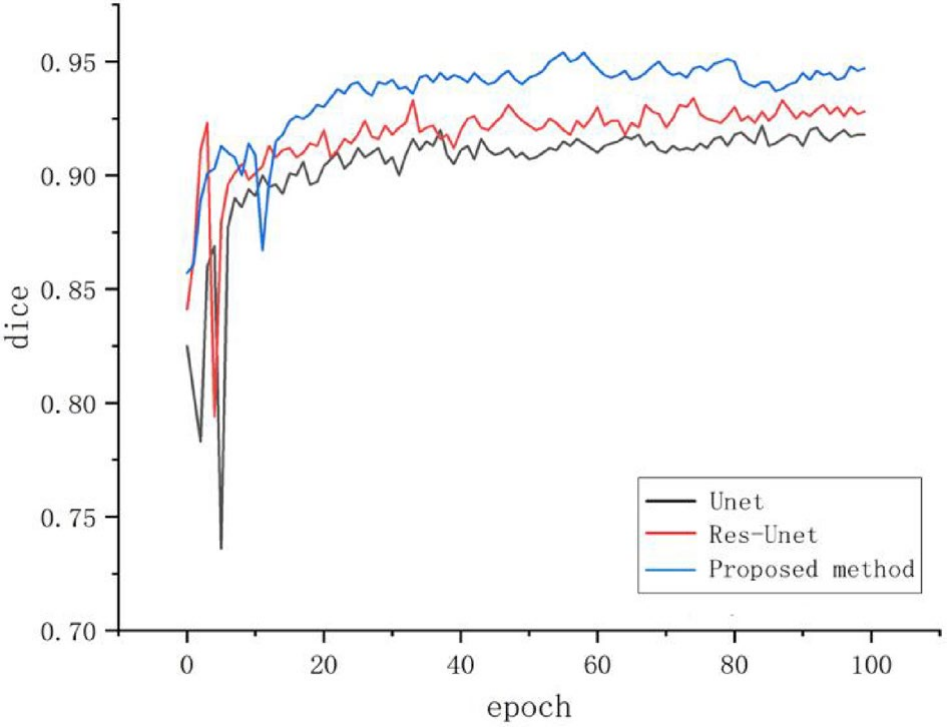
Dice curves of different model algorithms.

Figure 11 shows dice curves for different model algorithms. Dice loss is a measure function to evaluate the similarity of two samples, with a value range of 0–1. In the training process, we set the time to 100 times, and each epoch will be trained more than 400 times. Through comparison, we found that our model is higher than other models in terms of curve fluctuation and dice loss, which shows that our segmentation results have better performance.

**Figure 12:**
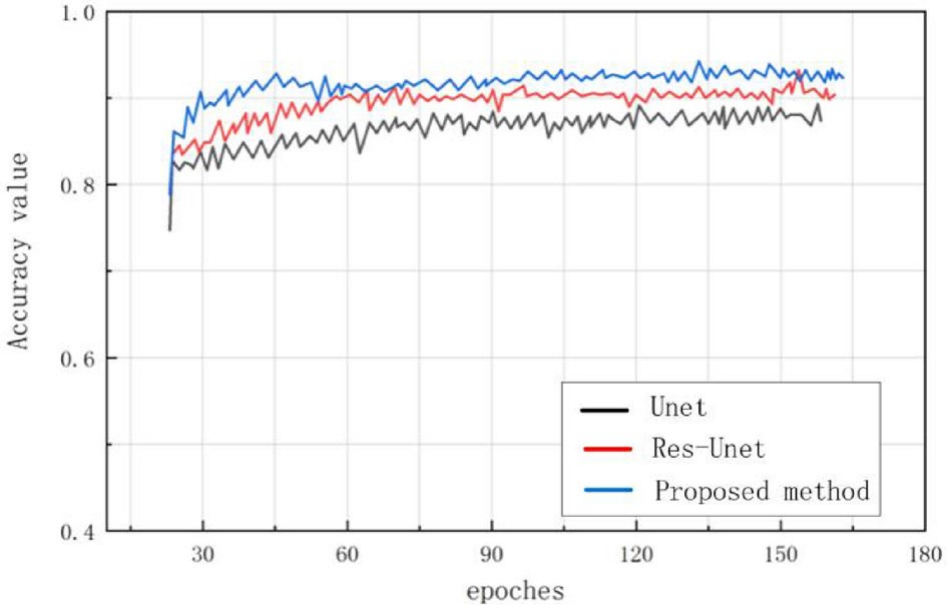
Comparison of Validation Accuracy.

Figure 12 shows that under the same training conditions, the segmentation accuracy of different algorithms varies with the number of iteration rounds in the state without preprocessing. It can be seen that our method is more stable in stable trends, with less accuracy fluctuations, reaching the highest accuracy, and stronger generalization ability.

**Figure 13:**
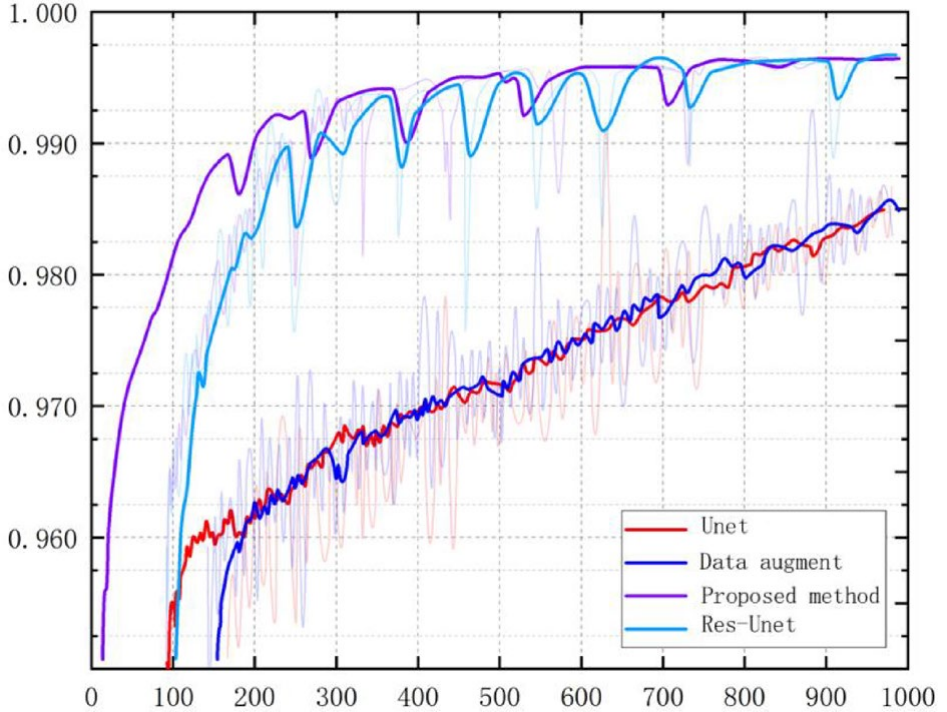
The accuracy rate of models.

The horizontal axis of Fig. 13 shows the number of rounds per 100 iterations of the network, while the vertical axis represents the accuracy or loss rate. It can be seen from the figure that our model has the best accuracy effect among the three training models, and the accuracy rate is also higher than other models. After a long time of testing, it tends to converge to a stable state, the convergence trend of other networks is slow. This shows that after the image is de-noised, it is segmented more accurately through the residual network, SE block and Unet network combined model segmentation.

**Figure 14:**
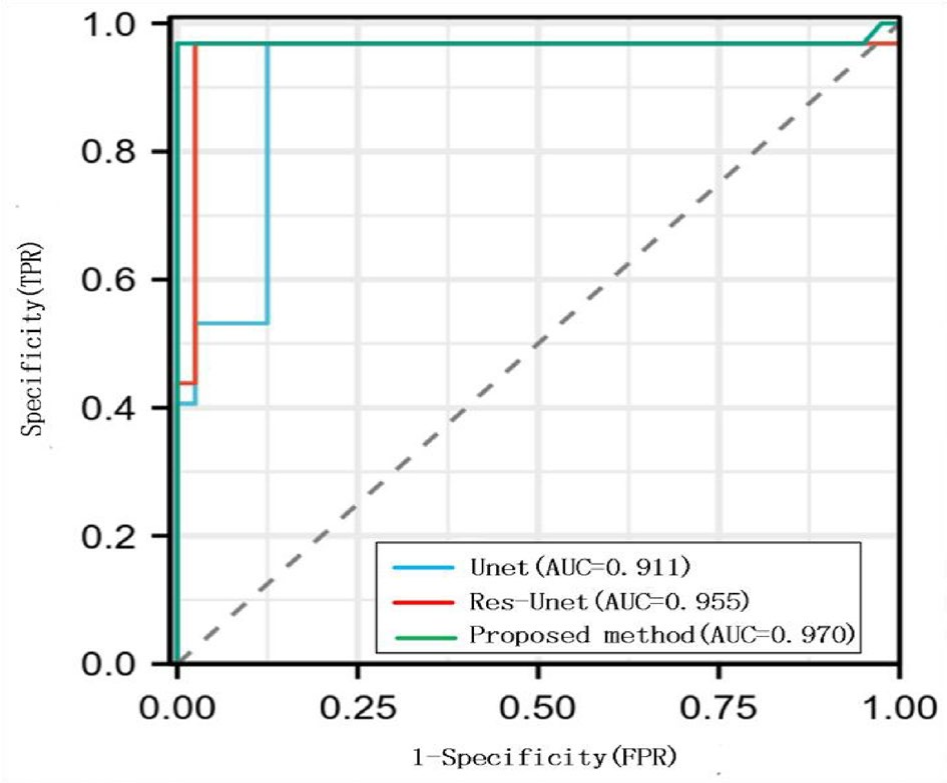
ROC curves of different model algorithms.

Figure 14 shows the ROC curves of different model algorithms. ROC curve is a curve reflecting the relationship between sensitivity and specificity. The X-axis of the abscissa is 1-specificity, also known as false positive rate. The closer the X-axis is to zero, the higher the accuracy is; The Y-axis of the ordinate is called sensitivity, also known as true positive rate (sensitivity). The larger the Y-axis is, the better the accuracy is. The area under the ROC curve (AUC) is often used for test evaluation. The AUC value range is generally between 0.5 and 1. The closer the AUC is to 1, the better the diagnostic effect of this variable in predicting the outcome. It can be seen from the figure that the Unet and Res-Unet algorithms are not as stable as proposed method in terms of trend change or comparison of AUC.

## Discussion

The segmentation of the liver is an important basis for the diagnosis and surgical treatment of liver cancer. However, in medical imaging, the shape and size of the liver change with the discontinuity of the region, and the texture changes are also very complex. In this work, we proposed image preprocessing and SE ResUnet to address the above issues. Specifically, we explicitly introduced preprocessing processes to enhance useful features in the image and help train the model to better analyze image information. At the same time, we introduced SE self attention modules and ResNet residual blocks for detailed capture of feature information, which helps accurately detect and segment image data.

Pretreatment studies have shown that PCNN filtering can effectively eliminate noise and preserve image boundaries and structural information. For example, in preprocessing experiments, PCNN filtering can perform good qualitative analysis on pixels, with better performance than median filtering. In training and segmentation experiments, it has been shown that enriching features and channel information through the network is more conducive to improving segmentation performance. Introducing the SE attention module and residual network into the U-net model, and then introducing the preprocessed data information into the network module, can better train data, with IoU and Dice increasing from 88 and 90.4% to 93.6 and 93.5%, respectively (Figs. 9 and 10).

In this experiment, we also compared the training and segmentation performance of the proposed SE ResUnet with ResUnet and Unet models. In terms of training performance, among the 100 training stages, the trend of SE-ResUnet is more stable and the accuracy fluctuation is minimal, highlighting the better convergence of SE-ResUnet (Fig. 11). However, after image denoising, the SE-ResUnet model remains stable after long-term training and can segment images more accurately (Fig. 12). In terms of segmentation performance evaluation, all indicators of SE ResUnet are superior to other models (Table 2). In the testing and evaluation, we found that the diagnostic performance was good in terms of SE ResUnet prediction results (Fig. 13). Meanwhile, from the visual effects of segmentation in Figs. 7 and 8, SE-ResUnet performs better in liver segmentation details than other classic models.

In this work, we used data provided by Hainan Provincial People's Hospital for model training and testing. Due to the presence of unclear edges and noise in the image itself, we introduced image preprocessing before training, enabling the PCNN algorithm to denoise and enhance the data in the image. We also designed and studied SE ResUnet based on the Unet model. In this way, it can not only deepen the network depth and extract more detailed features, but also prevent overfitting and improve the accuracy of the model. However, our research mainly focuses on the segmentation of two-dimensional planes, and further research should be conducted on the segmentation and reconstruction performance in three-dimensional backgrounds in future work.

## Conclusion

The study of liver cancer image and its accurate segmentation have always been of great significance for the timely and accurate analysis and detection of liver internal problems in clinical practice. This article first proposes an image preprocessing module using pulse coupled neural network (PCNN) algorithm for adaptive filtering to obtain normalized images. Then this study proposes an improved network SE-ResNet-Unet, which introduces the residual network module and the SE attention mechanism. The SE attention module with weighted characteristics is used as the input end of the residual block. After the output, it is inserted into the down-sampling and up-sampling process of Unet model by bilinear interpolation. From the experimental results, we can see that the method used can segment a clearer and relatively complete liver, reduce the impact caused by other inevitable operations such as machines, and improve segmentation accuracy and clarity. However, studying how to segment medical images more effectively and accurately is not an easy problem. This not only requires accurate evaluation of the heterogeneity of liver tumors, but also requires a large amount of annotated data, which is a limitation of our research. Therefore, in future research, we should continue to consider how to improve network models to improve performance, the advantages of multi model fusion algorithms in segmentation, and how to train in situations where labeled data is difficult to obtain.

## Data Availability

The datasets generated and/or analysed during the current study are not publicly available due [REASON WHY DATA ARE NOT UBLI] but are available from the corresponding author on reasonable request.

## References

[1] Zhou K, Song Z, Rostomian N (2023). Association of nativity with survival among adults with hepatocellular carcinoma. J. Natl. Cancer Inst..

[2] Hwang YJ, Bae JS, Lee Y (2023). Classification of microvascular invasion of hepatocellular carcinoma: Correlation with prognosis and MR imaging. Clin. Mol. Hepatol..

[3] Samantha AA, Aiwu RH (2020). Immuno-oncology for hepatocellular carcinoma: The present and the future. Clin. Liver Dis..

[4] Zhang Z, Zhang N, Cheng G (2023). Application of three-dimensional multi-imaging combination in brachytherapy of cervical cancer. Radiol. Med..

[5] Din M, Agarwal S, Grzeda M (2023). Detection of cerebral aneurysms using artificial intelligence: A systematic review and meta-analysis. J. NeuroInterv. Surg..

[6] Wu IC, Syu H, Jen CP, Lu M (2018). Early identification of esophageal squamous neoplasm by hyperspectral endoscopic imaging. Sci. Rep..

[7] de Godoy LL, Chawla S, Brem S (2023). Taming glioblastoma in 'real time': Integrating multimodal advanced neuroimaging/AI tools towards creating a robust and therapy agnostic model for response assessment in neuro-oncology. Clin. Cancer Res..

[8] Tan Y, Zhao SX, Yang KF (2023). A lightweight network guided with differential matched filtering for retinal vessel segmentation. Comput. Biol. Med..

[9] Gao, Z., Zong, Q. & Wang, Y. *et al.* Laplacian salience-gated feature pyramid network for accurate liver vessel segmentation. *IEEE Trans. Med. Imaging.*1–1 (2023, Online ahead of print). 10.1109/TMI.2023.3273528 37145950

[10] Tsai PC, Lee TH, Kuo KC (2023). Histopathology images predict multi-omics aberrations and prognoses in colorectal cancer patients. Nat. Commun..

[11] Xu, J., Guo, J. & Yang, H. Q. *et al.* Preoperative contrast-enhanced CT-based radiomics nomogram for differentiating benign and malignant primary retroperitoneal tumors. *Eur. Radiol.* (2023, Online ahead of print.). 10.1007/s00330-023-09686-x 37148350

[12] Liu Y, Hong Y, Lu Z (2021). An optimized pulse coupled neural network image de-noising method for a field-programmable gate array based polarization camera. Rev. Sci. Instrum..

[13] Zhu SS, Wu Y (2019). Unsymmetric trimmed median filter method based on improved PCNN decision. Instrum. Tech. Sens..

[14] Fan XN, Yan W, Shi PF (2019). PCNN simplified model combined with fast adaptive bilateral filtering crack image denoising algorithm. Foreign Electron. Meas. Technol..

[15] Liu Y, Chen S (2017). Review of medical image segmentation method. Electron. Sci. Technol..

[16] Gao JQ, Wang BB, Wang ZY, Wang YF, Kong FZ (2020). A wavelet transform-based image segmentation method. Optik Int. J. Light Electron Opt..

[17] Ren CL, Wang N, Zhang Y (2020). Summary of medical image segmentation methods. Netw. Secur. Technol. Appl..

[18] Wang GL, Sun Y, Wei BZ (2022). Systematic review on graph deep learning in medical image segmentation. Comput. Eng. Appl..

[19] Deepak P, Suyash B, Utku K (2022). Recent advancement in cancer diagnosis using machine learning and deep learning techniques: A comprehensive review. Comput. Biol. Med..

[20] Peng J, Luo HY, Zhao GS (2021). Survey of medical image segmentation algorithm in deep learning. Comput. Eng. Appl..

[21] Sidra G, Muhammad SK, Asima B (2022). Deep learning techniques for liver and liver tumor segmentation: A review. Comput. Biol. Med..

[22] Ali G, Renata R, Mohammadmehdi S, Dalibor Š (2022). Cell segmentation from telecentric bright-field transmitted light microscopy images using a Residual Attention U-Net: A case study on HeLa line. Comput. Biol. Med..

[23] Wang Y, Li YZ, Lai QG, Li ST, Huang J (2022). RU-Net: An improved U-Net placenta segmentation network based on ResNet. Comput. Methods Programs Biomed..

[24] Xie XW, Zhang WD, Wang HD (2021). Dynamic adaptive residual network for liver CT image segmentation. Comput. Electr. Eng..

[25] Jiang HY, Shi TY, Bai ZQ (2019). AHCNet: An application of attention mechanism and hybrid connection for liver tumor segmentation in CT columes. IEEE Access.

[26] Sun Q, Dai MY, Lan ZY, Cai F, Wei LF, Yang CC, Chen RQ (2022). UCR-Net: U-shaped context residual network for medical image segmentation. Comput. Biol. Med..

[27] Liu SP, Li YM, Zhou JJ (2020). Segmenting nailfold capillaries using an improved U-net network. Microvasc. Res..

[28] Cameron B, Jatin PS, Joseph KL (2022). Super U-Net: A modularized generalizable architecture. Pattern Recognit..

[29] Lv PQ, Wang JK, Zhang XY (2022). An improved residual U-Net with morphological-based loss function for automatic liver segmentation in computed tomography. Math. Biosci. Eng..

[30] Li ZM, Yang L, Shu LQ (2022). Research on CT lung segmentation method of preschoolb children based on traditional image processing and ResUnet. Comput. Math. Methods Med..

[31] Lin YC, Lin CH, Lu HY (2019). Deep learning for fully automated tumor segmentation and extraction of magnetic resonance radiomics features in cervical cancer. Eur. Radiol..

[32] Zhang DW, Huang GH, Zhang Q (2020). Exploring task structure for brain tumor segmentation from multi-modality mr images. IEEE Trans. Image Process..

[33] Zhang ZA, Wu CD, Sonya C, Dermot K (2020). DENSE-INception U-net for medical image segmentation. Comput. Methods Programs Biomed..

[34] Lou, A., Guan, S. Y. & Loew, M. DC-UNet: Rethinking the U-Net architecture with dual channel efficient CNN for medical image segmentation. In *Medical Imaging Image Process*, 758–768 (2021).

[35] Zheng, S. X., Lu, J. C., Zhao, H. S. *et al.* Rethinking semantic segmentation from a sequence-to-sequence perspective with transformers. In *Computer Vision and Pattern Recognition*, 6881–6890 (2021).

[36] Feng SL, Zhao HM, Shi F (2020). CPFNet: Context pyramid fusion network for medical image segmentation. IEEE Trans. Med. Imaging.

[37] Li XM, Chen H, Qi XJ (2018). H-DenseUNet: Hybrid densely connected UNet for liver and tumor segmentation from CT volumes. IEEE Trans. Med. Imaging.

[38] Huang QH, Chen YD, Liu LZ (2020). On combining biclustering mining and AdaBoost for breast tumor classification. IEEE Trans. Knowl. Data Eng..

[39] Shanmugapriya S, Pravda JRP, Rabia N, Javier P (2022). Deep learning for image-based liver analysis: A comprehensive review focusing on malignant lesions. Artif. Intell. Med..

